# Interpretable out-of-distribution diagnostics for enzyme stability prediction

**DOI:** 10.1093/bib/bbaf631.070

**Published:** 2025-12-12

**Authors:** Hongbo Chen, Li C Xia

**Affiliations:** Department of Statistics and Financial Mathematics, School of Mathematics, South China University of Technology; Department of Statistics and Financial Mathematics, School of Mathematics, South China University of Technology; Department of Statistics and Financial Mathematics, School of Mathematics, South China University of Technology

## Abstract

**Aim:**

Enzyme property predictors often degrade when deployed to new enzyme families whose sequence and label distributions differ from training data. Investigating how distribution shifts among enzyme families affect prediction error is crucial for building trustworthy protein engineering systems. Utilizing the latest learning theory [1] and accompanying DataShifts algorithm (https://github.com/DataShifts/datashifts/), we present a shift-aware diagnostic workflow that quantifies distribution shift across enzyme families and turns cross-family generalization error into an interpretable quantity.

**Methods:**

On the Kaggle Novozymes enzyme stability dataset, which contains nearly 10,000 samples spanning 180 enzyme families, we treat the 60 enzyme families with the largest pretraining errors as test domains with distribution shifts, and train the MLP model on the remaining enzyme families. For each test enzyme family, we produces a rigorous risk bound estimate and a bound decomposition that indicates whether performance is limited primarily by changes in sequence distribution (covariate shift) or by shifts in the sequence–label relationship (concept shift).

**Results:**

On the training enzyme families, the model’s MAE for predicting enzyme melting temperature is 2.70, whereas across the test families the average MAE reaches 5.74. Across test enzyme families, our interpretable risk bound tracks the actual test error closely, with a correlation of 93.7%. For the top 10 enzyme families with the largest errors, the average MAE reaches 12.38; the contributions of covariate shift and concept shift to the risk bound are 5.20 and 12.53, respectively.

**Conclusion:**

The primary cause of the model’s large predictive errors on unseen enzyme families is concept shift. In this case, simply expanding the training enzyme families data is unlikely to help, and fine-tuning on the new enzyme families is advisable.

**References:**

1. Hongbo Chen, and Li Charlie Xia. ‘General and Estimable Learning Bound Unifying Covariate and Concept Shifts.’ International Conference on Machine Learning (ICML) Dataworld Workshop 2025.

